# Early Childhood Dental Caries, Mouth Pain, and Malnutrition in the Ecuadorian Amazon Region

**DOI:** 10.3390/ijerph14050550

**Published:** 2017-05-22

**Authors:** Marvin So, Yianni A. Ellenikiotis, Hannah M. Husby, Cecilia Leonor Paz, Brittany Seymour, Karen Sokal-Gutierrez

**Affiliations:** 1 Department of Social and Behavioral Sciences, Harvard T.H. Chan School of Public Health, 677 Huntington Avenue, Boston, MA 02115, USA; 2 Harvard School of Dental Medicine, 188 Longwood Avenue, Boston, MA 02115, USA; yianni_ellenikiotis@hsdm.harvard.edu; 3 University of California Los Angeles Fielding School of Public Health, 650 Charles E. Young Drive S., Los Angeles, CA 90095, USA; hmhusby@gmail.com; 4 Doctor en Odontología General, Quito, Ecuador; cecamila@yahoo.com; 5 Department of Oral Health Policy and Epidemiology, Harvard School of Dental Medicine, 188 Longwood Avenue, Boston, MA 02115, USA; brittany_seymour@hsdm.harvard.edu; 6 University of California, Berkeley-University of California San Francisco Joint Medical Program, University of California, Berkeley School of Public Health, 570 University Hall, MC 1190, Berkeley, CA 94720, USA; ksokalg@berkeley.edu

**Keywords:** early childhood caries, mouth pain, malnutrition, Ecuador, community-based intervention

## Abstract

Malnutrition and dental caries in early childhood remain persistent and intertwined global health challenges, particularly for indigenous and geographically-remote populations. To examine the prevalence and associations between early childhood dental caries, parent-reported mouth pain and malnutrition in the Amazonian region of Ecuador, we conducted a cross-sectional study of the oral health and nutrition status of 1407 children from birth through age 6 in the “Alli Kiru” program (2011–2013). We used multivariate regression analysis to examine relationships between severe caries, parent-reported mouth pain measures, and nutritional status. The prevalence of dental caries was 65.4%, with 44.7% of children having deep or severe caries, and 33.8% reporting mouth pain. The number of decayed, missing and filled teeth (dmft) increased dramatically with age. Malnutrition was prevalent, with 35.9% of children stunted, 1.1% wasted, 7.4% underweight, and 6.8% overweight. As mouth pain increased in frequency, odds for severe caries increased. For each unit increase in mouth pain frequency interfering with sleeping, children had increased odds for being underweight (Adjusted Odds Ratio (AOR): 1.27; 95% CI: 1.02–1.54) and decreased odds for being overweight (AOR: 0.76; 95% CI: 0.58–0.97). This relationship was most pronounced among 3–6 year-olds. Early childhood caries, mouth pain and malnutrition were prevalent in this sample of young children. Parent-reported mouth pain was associated with severe caries, and mouth pain interfering with sleeping was predictive of poor nutritional status. We demonstrate the utility of a parsimonious parent-reported measure of mouth pain to predict young children’s risk for severe early childhood caries and malnutrition, which has implications for community health interventions.

## 1. Introduction

The global “nutrition transition” from traditional diets to low-quality, processed, high-sugar, high-fat, and carbohydrate-dense food and beverages poor in micronutrients [1] has led to dramatic increases in child undernutrition and chronic disease, being overweight, and obesity, particularly in low- and middle-income countries [1,2,3]. Child malnutrition is the most common contributor to disability and death among children under age 5 in low-income countries, and an impediment to national economic growth [4,5,6]. While undernutrition rates have declined globally, progress has been inconsistent across countries, and millions of children continue to suffer from the adverse physical and cognitive consequences [2,7].

In addition to these commonly-cited diet-related disorders, a pandemic of childhood dental caries has emerged [8,9]. Caries is the most common chronic disease of childhood, affecting 60–90% of young children worldwide. Untreated caries can lead to chronic oral infection, mouth pain, malnutrition, and reduced educational potential persisting into adulthood [8,10,11]. In fact, the 2010 Global Burden of Disease Study found that untreated caries in permanent teeth was the most prevalent out of 291 conditions evaluated, accounting for a substantial worldwide health burden [10]. The relationship between poor nutrition and childhood caries is complex, and epidemiologic research strongly suggests it involves bi-directional causation and comorbidity [12,13,14]. The evidence also indicates that preventing and treating early childhood caries could lead to improvements in oral health, nutrition and overall health that could benefit children in the short- and long-term [2,15,16]. 

Ecuador, like other Latin American countries, has experienced a nutrition transition and a high prevalence of childhood caries [6,17,18]. While 29% of children under age 5 have stunted malnutrition according to the most recent World Bank estimates [17], indigenous and rural populations have higher rates of stunting, nearly double the national average and quadruple the rate in Latin America [19]. Furthermore, 5% of children are overweight, and the prevalence of being overweight has continuously increased in recent years—aligning with the progressively common phenomena of co-existent undernutrition and obesity worldwide [3,20]. These data echo research observing significant disparities across a range of health outcomes for indigenous compared to non-indigenous communities in this region [21].

To address malnutrition and childhood caries, the University of California, Berkeley—in partnership with the Ecuadorian Ministries of Health and Education, Rotary International, and the community of Pueblo Kichwa de Rukullakta—piloted a community-based nutrition and oral health promotion program, Alli Kiru (“Beautiful Teeth” in the Kichwa language; http://www.allikiru.org/index.html)—in a network of rural indigenous communities in the Ecuadorian Amazon. Ongoing studies of the program are testing the acceptability and efficacy of low-cost oral health and nutrition interventions conducted by trained Kichwa health volunteers and preschool teachers in collaboration with Ministry of Health dentists, and have to-date observed significant reductions in caries prevalence and caries-related malnutrition among participants [22]. 

Despite burgeoning evidence positing childhood caries as a contributor to poor nutritional outcomes [13,22,23], few studies have empirically examined the relationship between the two in young children. Considering these diseases as intrinsically entwined might benefit community-based prevention and health promotion efforts which often face resource limitations and cordon off interventions specific by disease, despite shared risk factors [24,25]. Thus, for the present study, our objective was to examine the relationships between early childhood caries, parent-reported mouth pain, and nutritional status among participating children in the Kichwa community.

## 2. Materials and Methods

### 2.1. Study Population and Data Collection

The study population, recruitment, and data collection procedures are described in detail elsewhere [22], and briefly reported in the present study. Alli Kiru invited all children six months to six years of age and their families, across 23 Kichwa communities, to participate in community-based mobile dental camps three times per year. Our study sample consisted of 1407 children and their parent/caregiver on their first visit to Alli Kiru from 2011 to 2013. A trained health volunteer fluent in Spanish or Kichwa administered interviews to parents who provided written consent to participate, following verbal explanation of the study. Children were provided a verbal explanation of the study, and provided verbal assent. Questions included demographic characteristics, nutrition practices (e.g., whether the child was breastfed), oral health practices (e.g., whether the child visited a dentist), presence and frequency of mouth/dental pain, and access to medical/dental care. Children’s weight and height measurement were conducted barefoot and in light clothes and, using a digital scale and stadiometer (Seca, Chino, CA, USA). Child dental exams were performed by a licensed dentist; Decayed, Missing and Filled Teeth (dmft) index scores [26] were recorded by visual and clinical examination. The institutional review board of the University of California, Berkeley, reviewed and approved the study protocol (2011-04-3178), as did the Pueblo Kichwa de Rukullakta leadership, provincial directors from the local preschool and infant care programs, and the Ecuadorean Ministry of Health.

### 2.2. Measures

To assess child mouth pain, parents were asked three questions regarding the frequency of mouth pain, specifically (1) mouth pain at any time, (2) mouth pain interfering with eating, and (3) mouth pain interfering with sleeping. Parents responded on a four-point Likert scale (Never, Occasionally, Frequently, Almost Always). These were subsequently dichotomized into no mouth pain (those who responded Never) vs. has mouth pain (combining Occasionally, Frequently, and Almost Always) for this analysis.

Severe early childhood caries has been defined as the number of decayed, missing, or filled teeth exceeding a specific threshold for given child’s age [27]. In recognition of calls for simplified caries indices [28,29] and our interest in measuring depth of caries and related pain, we categorized caries severity using a cutoff based on the depth of the caries, hereinafter defined as “severe caries” (SC): children who had any distinct cavitated carious lesion depth at either the dentin or pulpal level (D2 or D3).

Child nutrition status was recorded based on recent World Health Organization (WHO) growth reference standards for infants and young children according to sex [30]. This included Height-for-Age, Weight-for-Height, and Weight-for-Age, normally transformed into z-scores using WHO AnthroPlus software version 3.2.2 for children under 5, and WHO AnthroPlus version 1.0.4 for children ages 5–6 (Geneva, Switzerland). We recorded the prevalence of child under-nutrition and those who were over-weight in this sample, including stunting/shortness (5th percentile of height-for-age; ≤−2 z-score), being underweight (5th percentile of weight-for-age; ≤−2 z-score), wasting/thinness (5th percentile of weight-for-height; ≤−2 z-score), and being overweight (95th percentile of weight-for-height; ≥+2 z-score) [30].

### 2.3. Data Analysis

We computed bivariate statistics and then used bivariate and multivariate ordered logistic regression procedures to calculate maximum likelihood estimates of odds ratios (ORs) and 95% confidence intervals for (1) the association of parent-reported child mouth pain and severe caries, and (2) the association of parent-reported child mouth pain and malnutrition (i.e., undernutrition and being overweight). Since the natural history of dental caries is to increase in incidence and severity with ages from six months to six years of age, we generated age category-stratified simple and multivariable models for severe caries (SC) among children age six months to two years, and children age 3–6 years.

Covariates for multivariate models were based on theoretical potential to modify the association between exposure and outcome of interest based on the literature [11,31,32], and exploratory stepwise analyses comparing unadjusted to adjusted ORs after inclusion of potential covariates. *X*^2^ and Wald *F*-statistics were inspected to test for unadjusted associations and overall model fit, respectively. Missing data was handled using listwise deletion. All analyses were performed using STATA software version 14 (Manufacturer, College Station, TX, USA).

## 3. Results

### 3.1. Demographics

Of the 1407 children examined, participants seen for their first visit in each year had similar demographic characteristics (Table 1). Mothers had a mean age of approximately 30 years, 8.2 years of education, and an average of four children. The majority (82.9%) of the children were 3–6 years old. Households had a mean of 6.7 individuals, and approximately 13% cooked with wood as their main source of fuel (a common indicator of very low socioeconomic status in global health research).

### 3.2. Oral Health and Nutrition Measures

**Figure 1:**
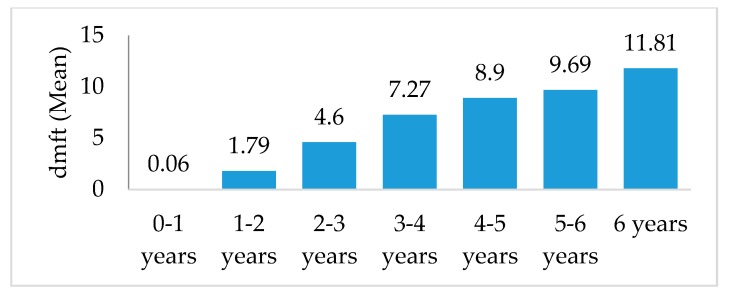
Mean Number of Decayed, Missing, or Filled Teeth (dmft) by age among first-visit children participating in Alli Kiru, 2011–2013.

In all, 65.4% of children had dental caries (dmft ≥ 1) and 44.7% had severe decay (D2 or D3). The mean number of decayed, missing and filled teeth increased steadily with age, with an average dmft of 0.06 among children less than one year, and 11.81 among children six years of age (i.e., over ½ of their baby teeth) (Figure 1). Mouth pain was present in 33.8% of children, with mouth pain interfering with eating and interfering with sleeping in 26.8% and 21.5% of children, respectively. Nearly a third of children in the sample were eating junk food (i.e., soda, sweets/candy, chips, or sweet ice pops) at least once a day. More than a third of children had ever been given a baby bottle (39.0%); among these children, milk or formula was the most common fluid given (43.2%), followed by sugar-sweetened beverages (29.1%) and water (16.5%). Furthermore, a third of these children sometimes fell asleep with the baby bottle in their mouth. About half of the mothers helped their child brush their teeth; and about half the of children had visited a dentist. Malnutrition was prevalent in our sample, with 35.9% of children stunted, 1.1% wasted, 7.4% underweight, and 6.8% overweight. 

### 3.3. Child Mouth Pain and Severe Caries

Univariate and multivariate analyses both demonstrated positive associations between measures of parent-reported mouth pain and severe caries (SC) (Table 2). Compared to children with no mouth pain and after adjusting for relevant covariates, as mouth pain increased in frequency, the odds for SC increased nearly three-times for each unit increase (Adjusted Odds Ratio (AOR) = 2.98; 95% Confidence Interval (CI): 2.36–3.75; *p* < 0.001); children whose mouth pain interfered with eating were more than twice as likely to have SC for each unit increase (AOR = 2.22; 95% CI: 1.79–2.76; *p* < 0.001) as were those whose mouth pain interfered with sleeping (AOR = 1.92; 95% CI: 1.53–2.41; *p* < 0.001). When stratified by age, all measures of parent-reported mouth pain were associated with SC among 3–6 year olds; no measures of mouth pain were associated with SC among 0–2 year olds.

### 3.4. Child Mouth Pain and Malnutrition

We did not observe significant associations for stunting, wasting, being underweight, or being overweight with unit increases in frequency of any mouth pain nor mouth pain interfering with eating. However, compared to children with no mouth pain, for each unit increase in frequency of mouth pain interfering with sleeping, children had increased odds for being underweight (AOR = 1.27; 95% CI: 1.02–1.54; *p* = 0.004) and reduced odds for being overweight (AOR = 0.76; 95% CI: 0.58–0.97; *p* = 0.032) (Table 3). 

Upon stratifying by age category, we found no significant risk for malnutrition associated with frequency of mouth pain among 0–2 year olds. However, among 3–6 year olds, compared to children with no mouth pain, as mouth pain interfering with sleeping increased in frequency, children had greater odds for being underweight (AOR = 1.45; 95% CI: 1.24–1.66; *p* = 0.004) and reduced odds for being overweight (AOR = 0.78; 95% CI: 0.67–0.94; *p* = 0.009). Significant risk for other malnutrition outcomes were not observed for mouth pain increase among 3–6 year olds.

## 4. Discussion

### 4.1. Prevalence of Caries and Mouth Pain

We observed a higher childhood caries prevalence and severity than some reports from Latin America writ large, Ecuador nationwide, and community studies of similar indigenous populations in Ecuador’s Amazon basin [18,34,35]. However, limited research exists on the prevalence and severity of caries among children under age 6, a critical period for the development of eating and oral hygiene habits. The high prevalence of mouth pain in our sample is of great concern, with clear associations established between poor oral health status, mouth pain and poor quality of life in childhood [15,36]. In children from birth to two years of age, the lack of association between parent-reported mouth pain and caries may be due to the challenge of distinguishing infant-toddler mouth pain from other discomfort and fussy behavior; and the predominance of brief, developmentally-appropriate episodes of mouth pain associated with tooth eruption and teething rather than chronic caries [37].

### 4.2. Prevalence of Malnutrition

The prevalence of malnutrition in our sample was high, and slightly different from that found in the most recent publications with national-level data we could find. Compared to national data on children less than five years old, our sample had higher rates of stunting (35.9% vs. 23.0%) and being underweight (7.4% vs. 6.2%) and lower rates of wasting (1.1% vs. 2.9%) and being overweight (6.8% vs. 7.5%) [38]. However, when compared to indigenous children in Ecuador, our sample had a lower rate of stunting (35.9% vs. 47.0%), wasting (1.1% vs. 2.8%) and being underweight (7.4% vs. 15.3%), and a higher rate of being overweight (6.8% vs. 3.4%) [17]. However, comparisons to the national average and indigenous children should be interpreted cautiously, as these data come from nearly one decade prior. Houck and colleagues documented a similar nutritional profile for young Kichwa children in this region, with a particularly concerning prevalence of stunting and low mean weight-for-age [39]. Additionally, they observed that the Kichwa ethnic group presented with the most significant child nutrition challenges compared to other indigenous groups across several communities, corroborating the urgency of action for this population.

### 4.3. Child Mouth Pain and Malnutrition

Mouth pain interfering with sleeping was the only exposure that remained significantly associated with child malnutrition after multivariable analysis. Without psychometric tests or mediation analysis, it is difficult to ascertain whether this is an artifact of the question (i.e., assessing child sleep disruption is the most reliable and internally-consistent way of asking about mouth pain), the role of sleep in influencing the relationship between tooth decay and malnutrition, or purely an indicator of severe tooth decay. Several studies have identified relationships between poor sleep quality with both being underweight and obesity in children. Plausible mechanisms include reduced sleep increasing energy expenditure; activating hormonal responses (i.e., leptin and ghrelin), which, in turn, affects appetite, food consumption, and activation of inflammatory processes that can contribute to chronic disease [40,41].

For 0–2 year olds, we did not find any measure of mouth pain associated with elevated odds for malnutrition, which is consistent with the lack of associations observed between perceived mouth pain and caries in this age group. However, among 3–6 year olds, mouth pain interfering with sleeping was a significant predictor of increased risk for underweight and decreased risk for being overweight. Vania et al. found similar results in 3–6-year-old Italian children, and proposed that severe caries likely forced a modification of child diet due to the pain of chewing [16]. Our study suggests that severe tooth decay in early childhood may be a substantial risk factor for malnutrition. Moreover, given how critical sleep is for healthful child development, this represents an important area for future research.

### 4.4. Implications for Addressing Oral Health and Malnutrition in Indigenous Communities

The high prevalence and severity of early childhood caries in our sample may reflect a shift in indigenous diets from native foods to non-traditional and cariogenic foods [6,11]. Over the past twenty years, malnutrition in Ecuador and across the Americas has decreased [7,18]. Tooth decay in 12-year-old children is reportedly lower, resulting from nutrition and agricultural education and food supplementation provided by multilateral global health agencies, improvements in oral health education, and caries prevention programs using fluoride [42]. However, these interventions have not diffused into the rural regions largely populated by indigenous communities, which may drive persistent disparities. Moreover, oral health interventions have not been adequately extended to the early childhood age group for which they would be most efficacious, leaving them at-risk for the most severe caries [21,43].

While dental healthcare providers are well-positioned to educate their patients and communities about the importance of healthful eating behaviors as essential for oral health [12], limitations in the extant health professions infrastructure in Ecuador—and Latin American more broadly—necessitates innovative solutions. The few randomized controlled trials that have examined the effect of treating caries for children’s anthropometric outcomes did not find significant improvements as a result of caries treatment [41,44], pointing to the need for a more comprehensive model of intervention that recognizes determinants at multiple ecological levels [3,14,25] and risk factors that are shared across malnutrition and dental caries [13,15,24]. We note that indigenous populations’ disproportionate malnutrition experience has emerged not due to genetics, but to broader structural drivers of inequality including geographic location, healthcare access, parental education, and health behaviors [17]. 

Community-based health promotion and disease prevention approaches, such as the *promotora* model, hold the potential to address this need, with a growing body of literature documenting promising efficacy for promoting child nutrition and preventing common childhood illnesses, inclusive of dental caries [24,25,45,46]. In their role as both health promoters and community members, they possess unique contextual and cultural knowledge that can prove invaluable for ensuring prevention efforts penetrate into medically-underserved regions, particularly in the context of obesogenic and cariogenic environments (e.g., rapid access to stores selling junk food). Since caregivers are so integral in shaping risk and protection for caries and malnutrition, they must be engaged in any community intervention and lay health workers are well-positioned to deliver these messages [17].

The parent-reported questions used in this study demonstrate the potential for routine demographic health surveys or rapid assessments by promotoras to identify children at risk for poor oral health and malnutrition, using parsimonious measures. This adds to a body of literature suggesting that caregiver perceptions of their child’s quality of life are a fairly reliable indicator of clinically-significant physical and oral health outcomes [32,36]. In collecting this data, tooth decay and malnutrition can be more readily detected and referred to care in settings where surveillance does not systematically occur. Among 3–6-year-olds, all mouth pain measures reliably predicted increased risk for severe caries, and mouth pain interfering with sleeping reliably predicted risk for being underweight. Acknowledging commonly-reported concerns with the consistent measurement of these conditions in children [29,47], we underscore the importance of additional research into easy-to-use, validated instruments with an eye towards building local capacity to prevent and mitigate community health issues.

### 4.5. Strengths and Limitations

Findings from this study should be considered in light of its limitations and strengths. Our study drew from a convenience sample of indigenous Kichwa communities, and may not be representative of other populations. In addition, the nature of the intervention limited our approach to cross-sectional analysis, given the heterogeneity of participants year-to-year and the intervention operating at both individual/family (e.g., individual dental screenings, family oral health education) and community levels (e.g., training of community health workers). This is unfortunately a widespread design flaw given the nascent body of research on this topic [41,44], and future investigations employing longitudinal and experimental are needed to better understand the cause-and-effect relationship.

Furthermore, we used an un-validated survey, administered in-person in conjunction with mobile dental screenings. Coupling of data collection with the intervention itself may have contributed to unmeasured social desirability bias, thereby resulting in underreporting of undesirable characteristics (e.g., child mouth pain); however, this is likely minimal given that we only included first-time visits in our analysis. Finally, our specific choice of index used to define caries makes it difficult to compare with other studies—a common challenge for the field [23]. We used dmft as our primary outcome measure; although it has historically been used for WHO caries diagnostic criteria, the International Caries Detection and Assessment System (ICDAS) may have been more appropriate to use. ICDAS was introduced to enable visual evaluation of patients’ tooth and restoration conditions with increased validity and reliability, and has since become commonplace in global oral epidemiologic surveys [29].

Nonetheless, our findings shed light on the high burden of dental decay and malnutrition in a population that is neglected in research—an indigenous community comprised of children under age 6. Our findings illuminate areas for future research—the psychometric testing of parent-reported measures for child mouth pain, as well as the mechanisms underlying disrupted sleep and undernutrition in children. Mediation analysis may be useful to confirm the degree to which the relationship between caries and malnutrition may be explained by mouth pain, and to identify other possible intervening variables. In light of emergent, yet promising, evidence regarding the feasibility and efficacy of community-wide programs addressing both oral health and malnutrition [13,22,41], this study reinforces the need for intervention developers to consider these conditions as deeply intertwined.

## 5. Conclusions

In a population with a high prevalence of severe dental caries and malnutrition, parent-reported mouth pain can reliably predict the presence of severe caries in young children, and parent-reported mouth pain interfering with sleeping can reliably predict poor nutritional status in children. This relationship seems to be most salient for children aged 3–6 years. To our knowledge, no previous study has demonstrated how a self-reported measure of mouth pain correlates with international standards of child malnutrition. Further research is needed to examine the comorbidity of dental caries and malnutrition, and the relationship between caries-related mouth pain and malnutrition across diverse populations. Dental caries and malnutrition are both multifactorial diseases with their own unique risk factors and with shared risk factors. Both can adversely affect children in their current stage of life and have poor outcomes for their future health, education and economic potential. There are some simple and effective steps that can be taken by maternal-child health professionals, promotoras and preschool teachers to identify young children at risk for severe caries and malnutrition, and to intervene in nutrition and oral health education, toothbrushing with fluoride toothpaste, fluoride applications, and treatment as needed to prevent the adverse consequences.

## Figures and Tables

**Table 1:** Demographic, child oral health, and child nutritional characteristics of first-visit children and their families participating in Alli Kiru, 2011–2013 (*N* = 1407).

Characteristic	2011 (*N* = 731)	2012 (*N* = 373)	2013 (*N* = 305)	Total
**Demographics**				
Maternal Age, Mean Years (SD)	30.0 (10.3)	30.3 (8.8)	30.0 (8.6)	30.1
Maternal Education, Mean Years Completed (SD)	6.9 (4.93)	8.4 (3.9)	9.3 (3.7)	8.2
Number of People per Household, Mean (SD)	7.0 (2.96)	6.7 (2.8)	6.5 (2.9)	6.7
Families Cooking with Wood Only ^a^, %	13.4	12.3	12.2	12.6
Number of Children per Mother, Mean (SD)	3.6 (2.6)	3.7 (2.4)	3.6 (2.3)	3.7
Child Age, %				
0–2 Years	15.5	17.6	18.3	17.1
3–6 Years	84.6	82.4	81.7	82.9
Child Sex, %				
Male	50.5	48.3	50.1	49.6
Female	49.5	51.7	49.9	50.4
**Child Oral Health and Oral Health Practices**				
Caries Prevalence by dmft Score ^b^, %				
Dmft ≥ 1	73.9	63.0	59.3	65.4
D2 or D3	47.5	41.3	45.2	44.7
D3	39.9	32.6	30.5	34.3
Parent-Reported Mouth Pain by Type, %				
No Mouth Pain	57.1	63.4	66.3	62.3
Any Mouth Pain	42.9	36.6	33.7	37.7
Mouth Pain Interfering with Eating	33.2	26.6	23.6	27.8
Mouth Pain Interfering with Sleeping	27.5	24.6	16.1	22.8
Number of decayed, missing and filled teeth (dmft), Mean (SD)				
0–1 years	0.0 (0.3)	0.1 (0.3)	0.1 (0.3)	0.1
1–2 years	1.7 (2.8)	1.7 (2.8)	2.0 (2.8)	1.8
2–3 years	4.5 (4.3)	4.7 (4.3)	4.6 (4.3)	4.6
3–4 years	7.2 (4.7)	7.3 (4.7)	7.3 (4.7)	7.3
4–5 years	8.9 (4.7)	8.8 (4.7)	9.0 (4.7)	8.9
5–6 years	9.5 (4.3)	9.0 (4.3)	10.6 (4.3)	9.7
6 years	11.2 (5.2)	12.0 (5.1)	12.2 (5.2)	11.8
Mothers Help with Child Toothbrushing, %	54.9	53.5	51.1	51.8
Child Ever Been to the Dentist, %	52.0	47.4	47.8	49.1
**Child Nutrition and Nutrition Practices**				
Prevalence of Malnutrition by Type ^c^, %				
Shortness/Stunting (HAZ < −2 SD)	35.8	34.1	37.8	35.9
Wasting/Thinness (WHZ < −2 SD)	1.5	1.1	0.7	1.1
Underweight (WAZ < −2 SD)	8.2	6.8	7.4	7.4
Overweight (WHZ > +2 SD)	5.6	11.7	3.1	6.8
Junk Food Consumption Frequency ^d^, %				
Every 2–4 Weeks	32.8	50.0	48.7	43.8
At Least Once a Week	58.9	55.0	43.6	52.5
At Least Once a Day	36.2	23.5	22.5	27.4
Child was Ever Breastfed, %	95.8	98.9	95.2	97.7
Child was Ever Bottlefed, %	40.3	41.6	37.4	39.0
What Child Drank in Baby Bottle ^e^, %				
Milk or Formula	39.9	39.7	52.1	43.2
Water	20.8	15.5	10.8	16.5
Sugar-Sweetened Beverage	28.5	29.6	32.2	29.1
Child Slept with Bottle ^f^, %				
Never	77.0	57.3	64.7	71.3
Sometimes	16.1	28.2	18.7	19.5
Frequently or Almost Always	6.9	14.6	10.6	9.1

dmft: Decayed, Missing, or Filled Teeth Score; HAZ: Height-for-Age Z-Score; WHZ: Weight-for-Height Z-Score; WAZ: Weight-for-Age Z-Score. ^a^ Cooking with wood as the primary source of fuel has been well-established as a valid consumption-based indicator of socioeconomic status in developing country settings and serves as a proxy for more traditional measures such as income in this study (see [33]). ^b^ Caries prevalence reported using dmft, an internationally-recognized system to determine the prevalence of dental caries. Components include decayed teeth, missing teeth due to caries, and filled teeth due to caries [26]. ^c^ Child growth indicators based on World Health Organization definitions from an international reference median value [30]. ^d^ Frequency of junk food consumption was assessed by asking parents, “How often does your child consume the following item?” for soda, sweets/candy, chips, and sweet ice pops. Respondents’ children were coded as having consumed junk food if any of those items were consumed in the given time period. ^e^ What the child drank in their baby bottle was assessed for parents who indicated that they gave their child a baby bottle at any point in time. Parents indicated whether the child drank water, milk, formula, lemonade, juice, coffee, natural juice, artificial juice, sugar water, chicha, and incaparina. Any beverage that was not water, milk, or formula was combined into a sugar-sweetened beverage category. ^f^ Whether the child slept with their baby bottle was assessed for parents who indicated that they gave their child a baby bottle at any point in time. Parents were asked, “How often did he/she fall asleep with the baby bottle in his/her mouth”?

**Table 2:** Adjusted odds ratios (AOR) and 95% confidence interval (95% CI) for severe caries (SC) according to parent-reported mouth pain.

Age Category	Any Mouth Pain (*N* = 476)		Mouth Pain Interfering with Eating (*N* = 378)		Mouth Pain Interfering with Sleeping (*N* = 303)	
	*N* (%)	AOR (95% CI)	*N* (%)	AOR (95% CI)	*N* (%)	AOR (95% CI)
All	329	2.59 ** (2.04–3.27)	206	2.23 ** (1.86–2.65)	162	1.92 ** (1.53–2.29)
0–2 year olds	74 (22.5)	2.52 (1.68–3.31)	46 (22.3)	1.40 (1.02–1.94)	36 (22.2)	1.58 (1.16–1.84)
3–6 year olds	255 (77.5)	2.77 ** (2.30–3.18)	160 (77.7)	2.08 * (1.67–2.59)	126 (77.8)	2.07 ** (1.74–2.42)

AORs adjusted for child age, child vaccination status, child breastfeeding history, child frequency of junk food consumption, and whether or not child had been to a dentist. * *p <* 0.05; ** *p <* 0.01.

**Table 3:** Adjusted odds ratios (AOR) and 95% confidence interval (95% CI) for malnutrition according to parent-reported mouth pain.

Measure of Malnutrition	Any Mouth Pain (*N* = 476)		Mouth Pain Interfering with Eating (*N* = 378)		Mouth Pain Interfering with Sleeping (*N* = 303)	
	*N* (%)	AOR (95% CI)	*N* (%)	AOR (95% CI)	*N* (%)	AOR (95% CI)
Stunting/Shortness Height-for-Age z ≤ −2	256	0.97 (0.67–1.24)	207	0.94 (0.65–1.23)	165	0.88 (0.62–1.12)
0–2 year olds	42 (16.4)	1.15 (0.87–1.32)	34 (16.4)	1.09 (0.87–1.30)	28 (17.0)	1.12 (0.86–1.36)
3–6 year olds	212 (82.8)	0.96 (0.73–1.12)	183 (88.4)	0.93 (0.72–1.14)	137 (83.0)	0.85 (0.61–1.05)
Wasting/Thinness Weight-for-Height z ≤ −2	27	0.86 (0.59–1.05)	28	0.63 (0.43–0.82)	21	1.13 (0.84–1.32)
0–2 year olds	6 (22.2)	0.93 (0.68–1.22)	6 (21.4)	0.82 (0.52–1.02)	4 (19.0)	1.47 (1.06–1.72)
3–6 year olds	21 (80.8)	0.66 (0.43–0.81)	22 (78.6)	N/A	17 (81.0)	N/A
Under-weight Weight-for-Age z ≤ −2	106	1.08 (0.94–1.20)	87	0.96 (0.72–1.15)	73	1.27 ** (1.02–1.54)
0–2 year olds	22 (20.8)	1.54 (1.21–1.79)	20 (23.0)	1.02 (0.84–1.13)	19 (26.0)	1.10 (0.91–1.32)
3–6 year olds	84 (78.5)	0.95 (0.77–1.11)	67 (77.0)	0.77 (0.53–0.95)	54 (74.0)	1.45 ** (1.24–1.66)
Over-weight Weight-for-Height z ≥ 2	87	1.06 (0.87–1.32)	56	1.10 (0.84–1.32)	44	0.76 * (0.58–0.97)
0–2 year olds	23 (26.4)	1.19 (0.87–1.32)	11 (19.6)	1.12 (0.82–1.32)	8 (18.2)	1.03 (0.67–1.37)
3–6 year olds	64 (73.6)	1.06 (0.72–1.38)	45 (80.4)	1.18 (0.86–1.34)	36 (81.8)	0.78 ** (0.67–0.94)

AORs adjusted for child age, child vaccination status, child breastfeeding history, child body mass index (BMI), and parental perception of child general and oral health. * *p* < 0.05, ** *p* < 0.01.
